# Virtual reality exoskeleton for post‐partum uterine tamponade balloon training: Impact on learning and operator satisfaction

**DOI:** 10.1002/ijgo.70385

**Published:** 2025-08-01

**Authors:** R. McConnell, A. McEvoy, T. Phillips, V. Ouranis, E. Mangina, F. M. McAuliffe

**Affiliations:** ^1^ UCD Perinatal Research Centre University College Dublin, National Maternity Hospital Dublin Ireland; ^2^ National Maternity Hospital Dublin Ireland; ^3^ The Magos Athens Greece; ^4^ University College Dublin School of Computer Science University College Dublin Belfield Co. Dublin Ireland

**Keywords:** medical education, postpartum haemorrhage, virtual reality

## Abstract

**Background:**

Virtual reality's (VR) use in medical education is increasing; however, traditional VR controllers lack real‐world dexterity. A post‐partum hemorrhage (PPH) is an obstetric emergency with significant maternal morbidity. The use of an intrauterine balloon reduces the need for further surgical interventions, but resident doctors might lack experience with balloon insertion.

**Objectives:**

This study evaluates whether a novel VR exoskeleton (VRE) improves medical students' and residents' uterine balloon insertion technique and learning experience compared to traditional didactic teaching.

**Methods:**

In a parallel‐group randomized controlled trial, clinical year medical students and residents were allocated to either: (i) a VRE group using an immersive VR tutorial with a haptic glove, or (ii) a control group receiving a standard slide‐based tutorial. All participants were assessed on the intrauterine balloon insertion technique and times using a pelvic model. Secondary outcomes included PPH knowledge (multiple‐choice questionnaire), confidence, side effects, and training acceptability. This trial was then compared to a previously published VR‐only cohort.

**Results:**

The VRE group showed slightly higher insertion technique scores and faster insertion times than the control group (*P* > 0.05). Both groups improved in confidence following training (*P* < 0.05), with no significant difference between arms. Participants in the VRE group reported higher satisfaction and felt more supported (*P* = 0.04). Exploratory comparison with the VR‐only group suggested improved technique with the exoskeleton, although demographic differences limit direct comparisons.

**Discussion:**

Virtual reality exoskeleton is a promising and well‐accepted tool for teaching obstetric skills. While both groups improved with training, VRE participants reported higher satisfaction and demonstrated modest gains in technique, supporting further investigation in larger trials.

## 1 BACKGROUND

Post‐partum hemorrhage (PPH), defined as greater than 500 mL blood loss within 24 h following childbirth,^1^ is a leading cause of global maternal mortality.^1^, ^2^ Globally, PPH remains a main cause of maternal death.^3^ PPH rates continue to rise and have increased by over 50% since 2005.^4^, ^5^ Ensuring our medical students and residents are trained in PPH management is critical, as early recognition and treatment improves maternal outcomes.^6^ Often, in emergencies, medical students, with or without prior PPH experience, are silent observers.^7^ Incorporation of simulation training into medical school or residents' training increases their PPH management confidence.^8^ Use of interdisciplinary team training, in general, leads to improved closed‐loop team communication and situational awareness.^9^, ^10^ Simulation training is more efficacious than traditional didactic lectures.^9^, ^11^ Conducting simulation training within a healthcare setting requires significant organization to arrange staff availability^12^, ^13^ and is often costly due to models and paying for staff.^13^, ^14^

Use of virtual reality (VR) within medical education has significant potential to enhance both theoretical learning^15^, ^16^, ^17^, and procedural skills.^18^ Traditional VR interaction methods, including handheld controllers or optical hand tracking, often fail to replicate real‐world dexterity accurately (which requires precise hand movements),^19^ reducing their effectiveness in training environments. The physical act of performing a skill in VR with haptic feedback increases a student's confidence prior to performing it in vivo.^20^, ^21^ Many VR systems rely on tracking methods that struggle with occlusions, environmental interference, and lack of tactile feedback.^22^ Medical education delivery has increasingly moved online since COVID‐19, with students lamenting the lack of “hands‐on” experience from in‐person teaching.^23^ Existing VR tracking systems often fall short of providing a seamless “hands‐on” experience due to sensor drift, limited tracking range, and calibration complexities.^24^, ^25^ This limits “traditional” VR's use in converting a two‐dimensional subject, such as anatomy, into a three‐dimensional subject,^17^ which has been incorporated across multiple specialities,^26^, ^27^, ^28^ including surgery^29^ and obstetrics and gynecology (O&G).^18^, ^30^

Advanced hand‐tracking technologies, such as exoskeletons, offer a solution by providing high‐fidelity motion tracking, precise joint articulation, and integrated haptic feedback. These systems allow users to interact with virtual environments in a manner that closely resembles real‐life procedures, improving both skill acquisition and retention.^19^ The Haptikos exoskeleton is designed to address these challenges and overcome the limitations of optical, magnetic, and inertial measurement unit tracking methods and to minimize interruption to the VR environment.^31^ The exoskeleton's enhanced accuracy and tactile feedback further augments the user experience in VR training, improving confidence in technical skills prior to their use in vivo.^18^, ^20^, ^21^, ^32^

Using exoskeletons in obstetric training might bridge the gap between traditional VR models and immersive VR‐based learning. Use of an intrauterine balloon is crucial to PPH management^6^ but also a relatively rare occurrence; hence, residents might be required to insert a uterine balloon in an emergency for the first time. Prompt recognition and treatment of PPH improves outcomes.^6^ An intrauterine balloon reduces the needs for further surgical intervention and/or maternal death in 85.9% of cases.^33^ Intrauterine balloons are particularly useful in PPH due to uterine atony,^33^ which accounts for 75% of PPHs.^4^ Prior training in intrauterine balloon insertion has been shown to improve maternal PPH outcomes.^34^ VREs might provide a more effective way for uterine balloon insertion training without the need to organize an entire simulation day. The aim of this study was to assess the use of VR exoskeleton (VRE) for uterine balloon training for medical students and resident doctors, specifically to assess the impact of VR training on the uterine balloon insertion technique. Additionally, this trial was compared to that of Dunlop et al.^18^ to assess if the use of the exoskeleton conferred additional benefit to VR alone.

## 2 METHODS

A multi‐center randomized control trial that recruited medical students from University College Dublin (UCD) and O&G resident doctors from the National Maternity Hospital, Dublin, Ireland, was conducted over a 4‐day period from Tuesday 10 to Friday 13 December, 2024. Ethical approval was obtained from the UCD Research Ethics Committee.

### 2.1 Participants

Participants were recruited from UCD's undergraduate (6‐year) or post‐graduate (4‐year) medical degree program and from the residents working in O&G at the National Maternity Hospital (NMH). All UCD medical students were recruited from the clinical years of the program. Residents recruited from NMH were either O&G residents (basic specialist training or higher specialist training) or general practice trainees undertaking a rotation through NMH. Residents were classified according to years post internship; <3 years, 3–5 years, or >5 years. Anyone aged under 18 years was excluded from participating. Participants within both UCD and NMH were recruited via email, word of mouth, and a secure online messaging app. A total of 20 participants were recruited across both sites. Informed written consent was obtained from all participants.

### 2.2 Randomization

**FIGURE 1:**
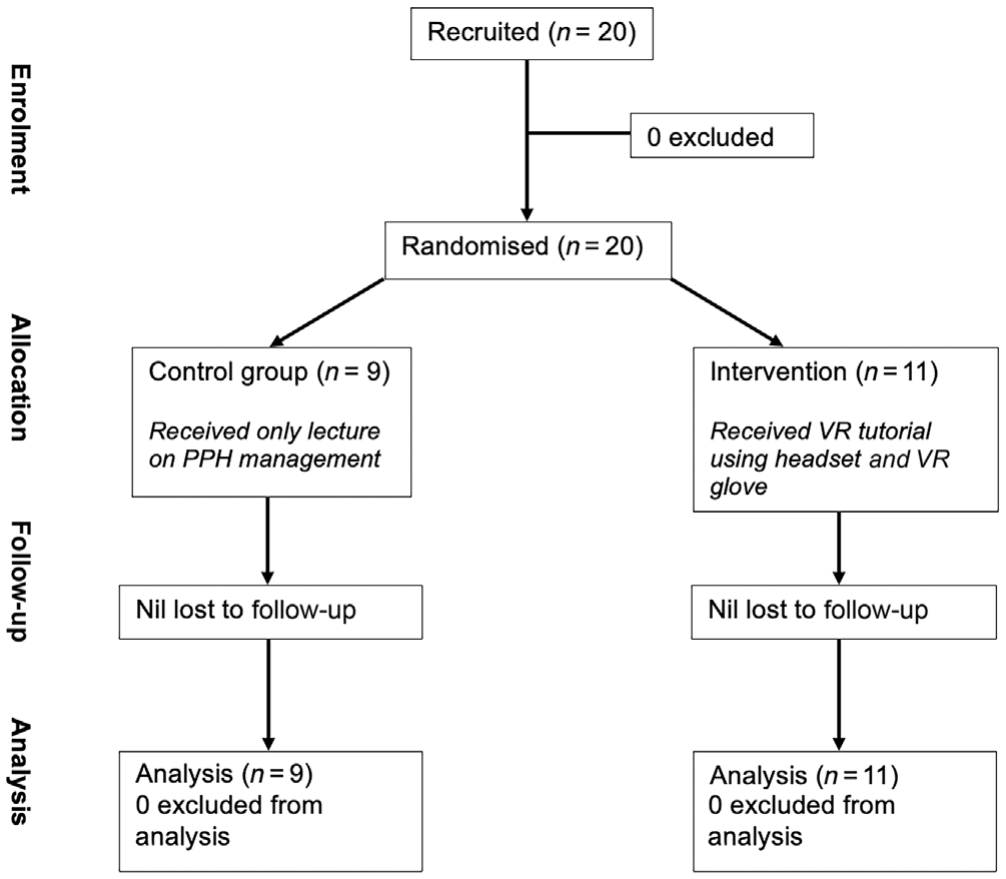
Randomization process within this study: Participants were given sequentially numbered envelopes with either randomization to the intervention or control. PPH, post‐partum hemorrhage; VR, virtual reality.

Figure 1 depicts the randomization process; participants were randomized to either the control or intervention as they enrolled. Participants were given sequentially numbered sealed brown envelopes with either control or intervention inside. A total of 50 randomization envelopes were created, with 25 for control and 25 for intervention. The researchers were not blinded to the participant or study group due to the nature of the study. Allocation was prospectively concealed to the participants, who were advised they could withdraw their consent at any stage, including after randomization. Prior to commencing either the VRE arm or control arm, participants answered a 10 question MCQ on PPH management and self‐assessed their confidence in inserting an intrauterine balloon using a Likert scale (1 = No confidence to 5 = Very confident).

### 2.3 Intervention and control groups

**FIGURE 2:**
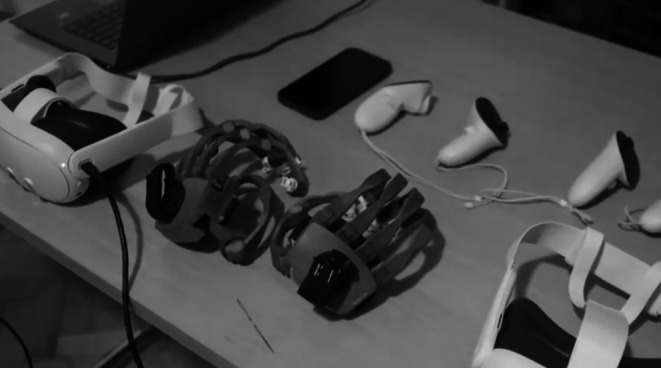
Virtual reality exoskeleton.

**FIGURE 3:**
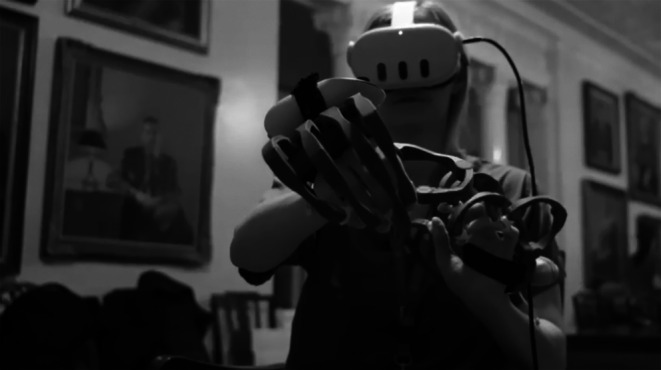
Virtual reality exoskeleton in use.

Participants assigned to the intervention group underwent a two‐part immersive virtual reality tutorial. For this tutorial, the Meta Quest 3 and Meta Quest 2 VR head‐mounted display was used along with a Haptikos VR Exoskeleton designed and developed by the UCD School of Computer Science and Magos, a Greek deep‐tech company (see Figure 2).^35^ Intervention participants received a virtual slide‐based presentation on PPH management and then placed an intrauterine balloon in an avatar, already placed in lithotomy. The Haptikos VR exoskeleton is a high‐precision hand‐tracking system that enhances the natural movement within the immersive environment (Figures 2 and 3). It integrates mechanical tracking sensors with proprietary algorithms to achieve submillimeter precision in all 24 degrees of freedom per hand.^22^ The Haptikos exoskeleton can be calibrated in one pose, reducing the need for complex setup.^36^ Tactile feedback allows the intervention group to experience material textures, allowing the intervention group to perform a uterine balloon insertion in an immersive VR environment.

The control arm received didactic teaching on PPH management, in printed slide format. The content of the slides was replicated from the first part of the VR tutorial. Prior to either VR exoskeleton or didactic teaching, both groups underwent a pre‐assessment multiple choice questionnaire (MCQ). Once the participants had completed the lectures, they were examined on the uterine balloon insertion technique and duration using a model pelvis, similar to an Objective Structured Clinical Exam. All participants were examined by one of three authors (RM, TP, or AMcE), who are residents within O&G. In both groups, between completion of the VR tutorial or lecture and assessment, participants underwent a repeat MCQ assessment and completed a student satisfaction survey on their method of teaching.

### 2.4 Outcomes

The main aim of this study was the uterine balloon insertion technique (marked from 1 to 10, with a point for each successful step). Other aims of the study included time for uterine balloon insertion, acceptability of the VR tutorial and exoskeleton versus the didactic lecture, side effects of VR, and overall student satisfaction. Confidence levels and improvements in participants' PPH knowledge were included as secondary outcomes. PPH knowledge was assessed using the MCQ score.

The results of this trial was compared to those of Dunlop et al.,^18^ a previously published trial assessing uterine balloon placement using traditional VR. This study used identical pre‐knowledge assessment, confidence, and satisfaction scores to this trial. Outcomes were analyzed between both intervention arms (VRE versus VR) and comparing outcomes between each intervention and respective groups.

### 2.5 Statistical analysis

Data collected was coded and entered into Microsoft Excel. All participants were given a study ID with no identifiers used for data collection. Statistical analysis was performed using Stata Version 18. Descriptive statistics were obtained for VR acceptability. χ^2^‐tests were used to analyze baseline demographic differences between groups. A paired *t*‐test was used to assess the change in confidence levels pre and post training for both the VRE and control. An independent *t*‐test was used to compare mean uterine balloon insertion scores between the VRE and control. A paired *t*‐test were used to compare mean pre and post MCQ scores. The Mann–Whitney *U*‐test was used to compare the distribution of intrauterine balloon insertion times.

The results of our study were compared to those of Dunlop et al.,^18^ an earlier trial using VR only to assess intrauterine balloon insertion in medical students. The VRE group of this study was compared to the VR‐only group of Dunlop et al.^18^ The study protocol, including acceptability questionnaires and assessment of learning, was identical to this study's. Independent *t*‐tests were used to compare the mean confidence scores and insertion scores.

## 3 RESULTS

### 3.1 Demographic data

Table 1 outlines the demographic data for our study cohort, plus the demographics of the VR‐only intervention and control arm of the trial by Dunlop et al.^18^ Within this trial, a total of 20 medical students/residents expressed an interest and subsequently participated. There was no significant difference between our control/intervention groups in respect to age, gender, or years post internship. The cohort used by Dunlop et al.^18^ was medical students only. No residents were enrolled, a major difference between the studies.

**TABLE 1:** Demographic data of virtual reality (VR) exoskeleton compared to the control (lectures), plus comparison of the VR‐only group versus control in Dunlop et al.^18^

	VR exoskeleton *N* = 11 (%)	Control (lectures) *N* = 9 (%)	*P*‐value	VR only *n* = 18 (%)	VR control (*n* = 16)	*P*‐value
Gender						
Male	4 (36)	3 (33)	0.88	2 (11)	7 (44)	0.03
Female	7 (64)	6 (67)		16 (89)	9 (56)	
Age (years)						
18–24	1 (9)	1 (11)	0.69	10 (56)	8 (50)	0.77
25–34	8 (73)	6 (67)		6 (33)	7 (44)	
35–45	1 (9)	2 (22)		2 (11)	1 (6)	
45+	1 (9)	0 (0)		0 (0.00)	0 (0)	
Stage of training (years post intern)						
Medical student	3 (27)	3 (33)	0.82	18 (100)	16 (100)	
<3	6 (55)	5 (56)		0 (0)	0 (0)	
3–5	1 (9)	1 (11)		0 (0)	0 (0)	
>5	1 (9)	0 (0)		0 (0)	0 (0)	

### 3.2 Uterine balloon placement confidence

Table 2 demonstrates the change in the mean pre‐training and post‐training confidence scores. Both VR and didactic lectures improved participants' confidence significantly for intrauterine balloon insertion (*P* = 0.003 and *P* = 0.049). There was no significant difference between the control and VR in the post‐training mean confidence level (*P* = 0.19).

**TABLE 2:** Pre‐ and post‐training confidence levels.

	Mean pre‐training confidence levels (SD)	Mean post‐training confidence levels (SD)	Mean difference (95% CI)	*P*‐value
Control (lectures) (*n* = 9)	2.00 (0.86)	2.66 (0.86)	0.66 (0.0009–1.33)	0.049
Intervention (VRE) (*n* = 11)	1.81 (1.07)	3.09 (0.53)	1.27 (0.74–1.80)	0.003

*Note*: 1 = No confidence, 2 = Little confidence‐ knows theory, 3 = Some confidence, would attempt with Senior Staff, 4 = Confident, know limitations & when to call for help, 5 = Very confident, can put in balloons.

Abbreviations: CI, confidence interval; MCQ, multiple‐choice questions; PPH, post‐partum hemorrhage; SD, standard deviation, VRE, virtual reality exoskeleton.

### 3.3 Pre‐ and post‐assessment scores

Table 3 demonstrates the intrauterine balloon insertion scores plus timing for each group. The VRE group demonstrated a slightly better technique (*P* = 0.54) and were faster at insertion (*P* = 0.31) than the control group. The VRE group had a higher pre‐training MCQ compared to the control (*P* = 0.04); however, there was no significant difference in the mean change in MCQ score between groups (*P* = 0.13).

**TABLE 3:** Uterine balloon assessment scores and MCQ scores for PPH knowledge assessment.

	Intervention (VRE) (*n* = 11)	Control (*n* = 9)	*P*‐value
Uterine balloon insertion assessment			
Mean score (SD)	8.63 (1.20)	8.22 (1.78)	0.54
Median time in minutes (Range)	3.4 (2–5)	4 (2–>5)	0.31
MCQ score			
Mean pre‐training (SD)	8.63 (1.12)	7.55 (1.5)	0.08
Mean post‐training (SD)	9.63 (0.67)	9.55 (0.88)	0.81
Mean change in MCQ (SD)	1.00 (1.26)	2 (1.58)	0.13

Abbreviations: MCQ, multiple‐choice questions; PPH, post‐partum hemorrhage; SD, standard deviation, VRE, virtual reality exoskeleton.

### 3.4 Participant satisfaction with virtual reality exoskeleton versus didactic lectures

Table 4 demonstrates the satisfaction of the participant with their modality of training. One student within the control group did not complete the satisfaction survey. Overall, participants were satisfied with their modality of teaching in both the intervention and the control. Participants within the VRE group felt significantly more supported during the VRE compared to the control group (1.18 [SD 0.40] vs. 2.00 [SD 1.19], *P* = 0.04).

**TABLE 4:** Participants satisfaction with VRE versus didactic lectures.

	Intervention (VRE) mean (*n* = 11) (SD)	Control mean (*n* = 8^a^) (SD)	*P*‐value
Teaching easy to follow/cues appropriate	1.81 (0.87)	1.37 (0.51)	0.21
Understood purpose of learning	1.63 (0.67)	1.37 (0.51)	0.37
Supported in learning process	1.18 (0.40)	2.00 (1.19)	0.04^a^
Tutorial designed for my knowledge level	1.90 (0.70)	1.73 (0.65)	0.18
Enhanced my understanding of PPH	1.54 (0.52)	1.75 (0.70)	0.47
Learning benefitted	1.54 (0.52)	1.75 (0.70)	0.47
Opportunity for further learning/feedback	1.80 (0.91)	2.25 (1.38)	0.42
Realistic experience	2.45 (1.12)	2.87 (1.45)	0.48
Recommend experience	1.63 (0.67)	2.00 (1.06)	0.37

*Note*: 1 = strongly agree, 5 = strongly disagree.

Abbreviations: PPH, post‐partum hemorrhage; SD, standard deviation, VRE, virtual reality exoskeleton.

^a^ One student within the control did not complete the satisfaction questionnaire.

### 3.5 Acceptability of the virtual reality exoskeleton glove

The majority of students agreed that the VR headset was comfortable to wear (*n* = 10 of 11, 91%) and easy to use (*n* = 10 of 11, 91%). Less than half of students reported side effects with the VRE (*n* = 5 of 11, 45%). Dizziness and disorientation were the most commonly reported side effects. No participant ceased the VRE training early due to side effects. Over 72% (*n* = 8 of 11) would recommend VRE as a learning tool, with 82% (*n* = 9 of 11) reporting that VRE was better than didactic teaching.

### 3.6 Comparison of the virtual reality exoskeleton versus virtual reality alone for uterine balloon training

Table 5 depicts the difference‐in‐differences (DiD) between the VRE/control in this study and the VR/control in Dunlop et al.^18^ When including all participants in both studies, the VR control group had the highest change in confidence score (+1.50, *P* < 0.001). Examining medical students alone, the VRE had the highest improvement in medical students' confidence score (+1.66, *P* = 0.03). Table 6 compares the DiD for insertion time and technique. Medical students using the VRE had a greater improvement in score and technique compared to the control; this improvement was larger for the VRE intervention group than the VR intervention group when compared to their respective controls. Reported side effects were lower in the exoskeleton group compared to the VR‐only group (*n* = 5/11, 45% vs. *n* = 11/18, 61%, *P* = 0.59).

**TABLE 5:** Comparison of the ‘difference‐in‐differences’ between our control (VRE) and intervention versus the intervention (VR) and control of Dunlop et al.^18^

	Mean pre‐training confidence score	Mean post‐training confidence score	Change in confidence	*P*‐value
All participants				
VRE (*n* = 11)	1.81	3.09	+1.28	0.003
VRE control (*n* = 9)	2.00	2.66	+0.66	0.04
VR (*n* = 17^a^)	1.29	2.76	+1.47	<0.001
VR control (16)	1.12	2.62	+1.50	<0.001
Medical students only				
VRE (*n* = 3)	1.00	2.66	+1.66	0.03
VRE control (*n* = 3)	1.33	2.66	+1.33	0.18
VR (*n* = 17^a^)	1.29	2.76	+1.47	<0.001
VR control (*n* = 16)	1.12	2.62	+1.50	<0.001

Abbreviations: VR, virtual reality; VRE, virtual reality exoskeleton.

^a^ One student did not complete the confidence score.

**TABLE 6:** Comparison for the ‘difference‐in‐differences’ between this study and Dunlop et al.^18^ for uterine balloon insertion time and insertion score.

	VRE (*n* = 11)	VRE control (*n* = 9)	Difference between VRE and control	*P*‐value	VR (*n* = 18)	VR control (*n* = 16)	Difference between VR vs. control	*P*‐value
All participants								
Mean insertion time (min)	3.4	4.0	−0.60	0.31	2.44	3.12	−0.68	0.02
Mean insertion score	8.63	8.22	+0.41	0.54	7.55	7.46	+0.09	0.73
	**(*n* = 3)**	**(*n* = 3)**			**(*n* = 18)**	**(*n* = 16)**		
Medical students only								
Mean insertion time (min)	2.5	4.33	−1.83	0.20	2.44	3.12	−0.68	0.02
Mean insertion score	8.3	7.3	+1.00	0.34	7.55	7.46	+0.09	0.74

Abbreviations: VR, virtual reality; VRE, virtual reality exoskeleton.

## 4 CONCLUSION

This randomized controlled trial evaluated the use of a novel VRE to enhance the intrauterine balloon insertion, a key technical skill in PPH management. From our study, the exoskeleton was highly acceptable to students, with 90.0% reporting it was easy to use and comfortable to wear. Students reported feeling more supported with the VRE compared to the lecture slides. In comparison with Dunlop et al.,^18^ our study cohort reported fewer side effects with the exoskeleton. Similar studies have reported side effect rates of up to 62%,^18^, ^30^ which is higher than the 45% in our cohort. Nearly three‐quarters of our cohort would recommend the exoskeleton and felt it improved their O&G learning; this is similar to other immersive VR studies.^16^ Limitations of haptic VR training include the inability to differentiate between different areas, as described in one dentistry trial, due to indiscriminate haptic feedback.^32^ Within this study, 86% of students felt that haptic VR was not a replacement for conventional teaching.^32^ The Haptikos exoskeleton has been designed specifically to overcome a lack of tactile feedback,^22^ allowing replication of real‐world dexterity by integrating mechanical tracking sensors to allow precision for all 24 degrees of freedom per hand and reducing occlusion and drift.^19^ This makes the exoskeleton more applicable to teaching practical skills.

Use of the VRE to perform a uterine balloon insertion on a VR avatar translated to a better insertion technique and increased confidence among medical students compared to VR alone. The VRE is a novel and suitable method for teaching practical skills, providing the material textures,^37^, ^38^ which is lacking when using VR handheld controllers alone.^22^ The ease of use and minimal set up might allow students to undergo simulation training for specific practical skills at a time that is convenient for them.^36^ While simulation will always be required, the VRE might provide a useful adjunct in allowing staff to practice practical skills with VR, leaving the simulation days for focusing on interdisciplinary communication skills.^8^, ^9^

Our study is a pilot study assessing the acceptability of the VRE, and demonstrating the potential uses for it within medical education. Its strength is that it examines a novel technology, the exoskeleton, which has not been extensively examined within the literature. The limitation of this study is the sample size; hence, further, larger studies are required in the future.

This real‐world dexterity of the Haptikos exoskeleton and its application to teaching a practical skill are demonstrated in the comparison between the exoskeleton and VR‐only groups. The groups who performed the uterine balloon training with the exoskeleton had a better uterine balloon technique compared to the VR‐only group. Previously, VR has been relatively limited to knowledge‐based teaching.^20^, ^39^ The use of the VRE demonstrates its potential for teaching practical skills such as uterine balloon insertion. It may be beneficial for skills which are relatively uncommon but often occur in emergencies.

A limitation of our study is its small sample size. The strength of this study include that it uses a novel VRE in teaching a practical skill. This use of VR has potential within medical education.

In summary, the VRE is an acceptable teaching tool, with participants preferring it to didactic teaching. The VRE training led to a higher increase in confidence and insertion scores within medical students compared to traditional VR training.

## AUTHOR CONTRIBUTIONS

R. McConnell: Running the trial, collecting data, data analysis & article write up. A. McEvoy: Study design, implementing the trial, data collection & article review. T. Phillips: Running the trial, collecting data & article review. V. Ouranis: Concept design, study conduction & creating exoskeleton. E. Mangina: Study design, sourcing funding, article review and trial supervision. F. M. McAuliffe: Study design, sourcing funding, article review and trial supervision.

## FUNDING INFORMATION

This research was supported by funding from the European Union's Horizon Europe FIDAL Field Trials beyond 5G, SNS Large Scale Trials and Pilots (LST&Ps) with Verticals program under grant agreement No. 101096146.

## CONFLICT OF INTEREST STATEMENT

None of the authors have no conflicts of interest to disclose.

## Data Availability

The data that support the findings of this study are available from the corresponding author upon reasonable reques.
